# Editorial: Antimycobacterial drug discovery: molecular therapeutics and target identification, Volume II

**DOI:** 10.3389/fphar.2023.1202287

**Published:** 2023-07-11

**Authors:** Hendra Gunosewoyo, Gert Kruger

**Affiliations:** ^1^ Curtin Medical School, Faculty of Health Sciences, Curtin University, Bentley, WA, Australia; ^2^ University of KwaZulu-Natal, Durban, KwaZulu-Natal, South Africa

**Keywords:** *Mycobacterium tuberculosis*, antimicrobial, structure-activity relationship, whole genome sequence (WGS), multi-drug resistance (MDR), drug repurposing

We are delighted to publish the second volume of the Research Topic: Antimycobacterial Drug Discovery—Molecular Therapeutics and Target Identification. The Research Topic continues the discussion with an emphasis on the identification of novel antimycobacterial compounds and, more broadly, antibacterial compounds isolated from natural products or derived synthetically. Studies on the mechanisms of action of these bioactive molecules provide insights into the design of safer therapeutics and antimicrobials for the treatment of multidrug-resistant infections.

The second volume begins with a review of the antimicrobial properties of phytochemicals by Khare et al. The authors provide a comprehensive summary of the effects produced by alkaloids, phenolic compounds, terpenes, and coumarins against drug-resistant pathogens, with a focus on the major determinants of antibiotic resistance, such as efflux pumps, cell permeability, and modification of the antibiotic targets. Rubio et al. report on the mechanism of cellular uptake of mycolactone, a lipid toxin produced by *Mycobacterium ulcerans*, from the circulating plasma Using a combination of dynamic light scattering and fluorescence spectroscopy, the authors demonstrate how mycolactone behaves in solution and how it interacts with serum albumin and lipoproteins in plasma. Monitoring plasma levels of complexes formed by mycolactone and albumin/lipoproteins could be useful for the early diagnosis of patients with Buruli ulcer disease. Wang et al. investigate the effect of the labdane diterpenoid andrographolide on the hemolytic activity of *S. aureus*. Andrographolide dose-dependently inhibits hemolytic activity by significantly downregulating the transcript levels of the *Hla* gene, which in turn is responsible for the expression of the crucial virulence protein Hla. Molecular dynamics simulations of Hla-andrographolide interactions provide valuable insights into the structural requirements for the observed activity and have the potential to design more potent andrographolide analogs. Hoffmann et al. demonstrate the repurposing potential of nitroxoline for the treatment of multidrug-resistant tuberculosis (MDR-TB). Originally approved for the treatment of urinary tract infections, nitroxoline was tested in a panel of MDR-TB cells and found to have MIC values of 4–8 mg/L. Finally, Korycka-Machala et al. report the synthesis of thiosemicarbazide-based compounds and their structure-activity relationship against *Mycobacterium tuberculosis.* The authors demonstrate the efficacy of these compounds in inhibiting the growth of intracellular *M. tb* and reducing biofilm formation. The selection of resistant mutants and their whole genome sequencing identified the *mmpR5* gene as a target for these analogs.

Taken together, the second volume of this Research Topic: “Antimycobacterial Drug Discovery: Molecular Therapeutics and Target Identification”, provides recent advances in the development of antibacterial and antimycobacterial agents. We acknowledge the contributions of all the authors that made this research volume possible.

